# Application of drug-induced sleep endoscopy in infants with dynamic upper airway collapse

**DOI:** 10.3389/fped.2025.1614895

**Published:** 2025-09-25

**Authors:** Qing Wei, Ruimin Yang, Xun Chen, Xiang Yi, Jing Liu, Yan Li

**Affiliations:** ^1^ Department of Pediatrics, The First Affiliated Hospital of Guangxi Medical University, Nanning, China; ^2^ Department of Anesthesiology, The First Affiliated Hospital of Guangxi Medical University, Nanning, China; ^3^ Department of Otolaryngology—Head and Neck Surgery, The First Affiliated Hospital of Guangxi Medical University, Nanning, China

**Keywords:** drug-induced sleep endoscopy, infant, dynamic upper airway collapse, laryngomalacia, pharyngeal airway collapse, midazolam

## Abstract

**Objective:**

The study aimed to evaluate the utility and safety of drug-induced sleep endoscopy (DISE) in infants with suspected dynamic upper airway collapse.

**Methods:**

Infants with suspected dynamic upper airway collapse were enrolled in the study. All subjects developed clinical symptoms within the first year of life. Each subject underwent both awake endoscopy (AE) and DISE. Endoscopic findings and sedation strategies for DISE were recorded. The diagnostic rate of dynamic upper airway collapse was compared between the DISE and AE. Adverse events during DISE were also recorded.

**Results:**

(1) A total of 21 cases were included. The median age at the time of bronchoscopy was 4.0 months. (2) For the cases beyond neonatal age (*n* = 18), 16 (88.9%) received midazolam only, and 2 (11.1%) received midazolam combined with dexmedetomidine. For the neonates (*n* = 3), two (66.7%) received 10% chloral hydrate only, and one (33.3%) received 10% chloral hydrate combined with phenobarbital. (3) Six cases (28.6%) were diagnosed under both AE and DISE, whereas 15 cases (71.4%) were diagnosed under DISE only. The diagnostic rate was significantly higher under DISE than that under AE (100.0% vs. 28.6%, *P* < 0.01) in the cases with dynamic upper airway collapse. Of the cases with laryngomalacia, 3 cases (18.7%) were diagnosed under both AE and DISE, whereas 13 cases (81.3%) were diagnosed under DISE only. The diagnostic rate was significantly higher under DISE than that under AE (100.0% vs. 18.7%, *P* < 0.01) in the cases with laryngomalacia. Of the cases with tongue base collapse, all (100.0%) were diagnosed under both AE and DISE. Of the cases with retropalatal and hypopharynx collapse, all (100.0%) were diagnosed under DISE only. (4) One case (4.8%) developed a hypoxic episode during DISE, which was resolved by the pressurized facial mask-assisted ventilation.

**Conclusions:**

DISE was found to be a feasible and safe procedure in infants with suspected dynamic upper airway collapse. Compared with AE, DISE significantly improved the diagnostic rate of laryngomalacia and appeared to be a more reliable method to diagnose pharyngeal airway collapse, especially retropalatal and hypopharynx collapse.

## Introduction

Drug-induced sleep endoscopy (DISE) is a medical procedure used to identify sites of upper airway obstruction and collapse in patients with sleep-related respiratory issues, such as obstructive sleep apnea (OSA) syndrome (1). When an endoscopy is performed during pharmacologically induced sleep, upper airway blockages and collapse can be detected, and their types, levels, and patterns can be determined (1). DISE was first described by Croft and Pringle in 1991. It has been widely adopted and is frequently utilized to evaluate adults with OSA, which can help make the treatment decision and predict surgical outcomes (2). In children, especially in infants, DISE is still in the exploratory stage, and the indications, sedation regimen, endoscopy protocol, and the interpretation of DISE findings remain controversial (2, 3). In this study, we conducted DISE in infants with suspected dynamic upper airway collapse to evaluate the utility and safety of DISE in infants.

## Subjects and methods

### Subjects

All subjects were enrolled from the Department of Pediatrics at the First Affiliated Hospital of Guangxi Medical University from August 2023 to April 2025.

Children with a suspected diagnosis of dynamic upper airway collapse who underwent bronchoscopy during the first year of life were enrolled. Children who developed clinical signs of dynamic upper airway collapse during the first year of life but underwent bronchoscopy later were enrolled. A suspected diagnosis of dynamic upper airway collapse was made in infants with clinical manifestations of stridor, noisy breathing, snoring, and suprasternal retraction (4, 5). A definitive diagnosis of dynamic upper airway collapse was confirmed by endoscopy.

The exclusion criteria were as follows: (1) subjects requiring non-invasive or invasive mechanical ventilation; (2) subjects with choanal atresia or significant nasal cavity stenosis that prevented passage of a flexible bronchoscope (outer diameter 2.8 mm); and (3) subjects with tracheobronchial anomalies resulting in excessive airway stenosis or severe pneumonia.

### Methods

#### Procedure of DISE

Informed consent was obtained from the legal guardians of the subjects. The infants underwent a pre-operative fasting period of 2 h for liquids and 6 h for solids. Routinely, 4 mL of 2% lidocaine inhalation and 0.01–0.02 mg/kg of atropine were administered subcutaneously 30 min before bronchoscopy. In the operating room, the infants were placed in the supine position, and heart rate (HR), oxygen saturation (SpO_2_), and blood pressure (BP) were monitored. Bronchoscopy was performed under spontaneous breathing with unilateral nasal catheter oxygen inhalation. Firstly, bronchoscopy was performed in an awake state without the use of any sedative or anesthetic drugs. Local anesthesia was induced by spraying 2% lidocaine hydrochloride solution over the nasal cavity, avoiding the nasopharynx, oropharynx, hypopharynx, or larynx. In addition, tetracaine (tetracaine 1% SDU Faure; Novartis, Basel, Switzerland) was applied to the surface of the bronchoscope as a local anesthetic. The flexible bronchoscope (BF-XP290; Olympus, or QG-3320; SeeSheen) was inserted through the nasal cavity. Endoscopic findings of the palate/velum, lateral oropharyngeal wall, tongue base, and supraglottic larynx were recorded in the awake state. If obstruction was present, the site, pattern or shape, and severity of obstruction were recorded. Next, the infants were given adequate sedation. For the cases beyond neonatal age, sedation strategies included 3–4 µg/kg of intranasal dexmedetomidine and/or 0.1 mg/kg of midazolam (not exceeding 0.3 mg/kg) or 2–3 mg/kg of propofol via intravenous push (2, 6, 7). For the neonates, sedation strategies included 0.5–1.0 mL /kg of 10% chloral hydrate enema either alone or combined with 5 mg/kg of phenobarbital via intravenous push (6). The level of sedation allowed for flexible endoscopy without patient reactivity or awakening (2). A thin bronchoscope with an outer diameter of 2.8 mm was used in all cases. Endoscopic findings, as mentioned above, were recorded under adequate sedation. Finally, the flexible bronchoscope was passed through the glottis to the lungs for routine examination of the trachea and bronchi. Topical anesthesia with 1 mL of 2% lidocaine was administered in the glottis, trachea, and the main bronchus. During the procedure, when SpO_2_ dropped below 85%, bronchoscopy was terminated, and oxygen flow was increased, or pressurized mask ventilation was applied. When SpO_2_ returned above 95%, bronchoscopy was resumed.

#### Diagnostic criteria for pharyngeal airway collapse, laryngomalacia, and dynamic upper airway collapse under the endoscopy

Pharyngeal airway collapse (PAC) was defined as a reduction of more than 50% in the pharyngeal internal diameter during inspiration, causing airway obstruction (8). Based on the collapse region, PAC was classified as retropalatal collapse, hypopharynx collapse, or tongue base collapse (1, 9). Multilevel collapse was defined as the presence of two or more collapse regions simultaneously. Laryngomalacia was defined as an inward collapse of the supraglottic structures into the glottis during inspiration, causing airway obstruction (10). Laryngomalacia was classified as type I (redundant arytenoid mucosa), type II (short aryepiglottic fold with curled epiglottis), type III (epiglottic collapse), and mixed type with the presence of two or more types simultaneously (10). Dynamic upper airway collapse was defined as the presence of PAC and/or laryngomalacia. The diagnosis was made after the deliberation of two endoscopists.

#### Data collection

Clinical data were recorded by collecting data on medical history, including the age of onset and clinical manifestations during wakefulness and sleep. Endoscopic findings in the awake state and under adequate sedation were recorded as mentioned above. The sedation protocol and sedation-related complications were also recorded.

#### Statistical analysis

Statistical analysis was performed using SPSS 20.0 software. Measurement data are expressed as medians (25th–75th percentile). Counting data are expressed as count or percentage. For the categorical variables, between-group comparisons were performed using the *χ*^2^ test or Fisher’s exact test. A two-sided *P*-value of <0.05 was considered statistically significant.

## Results

### General information

**Figure 1:**
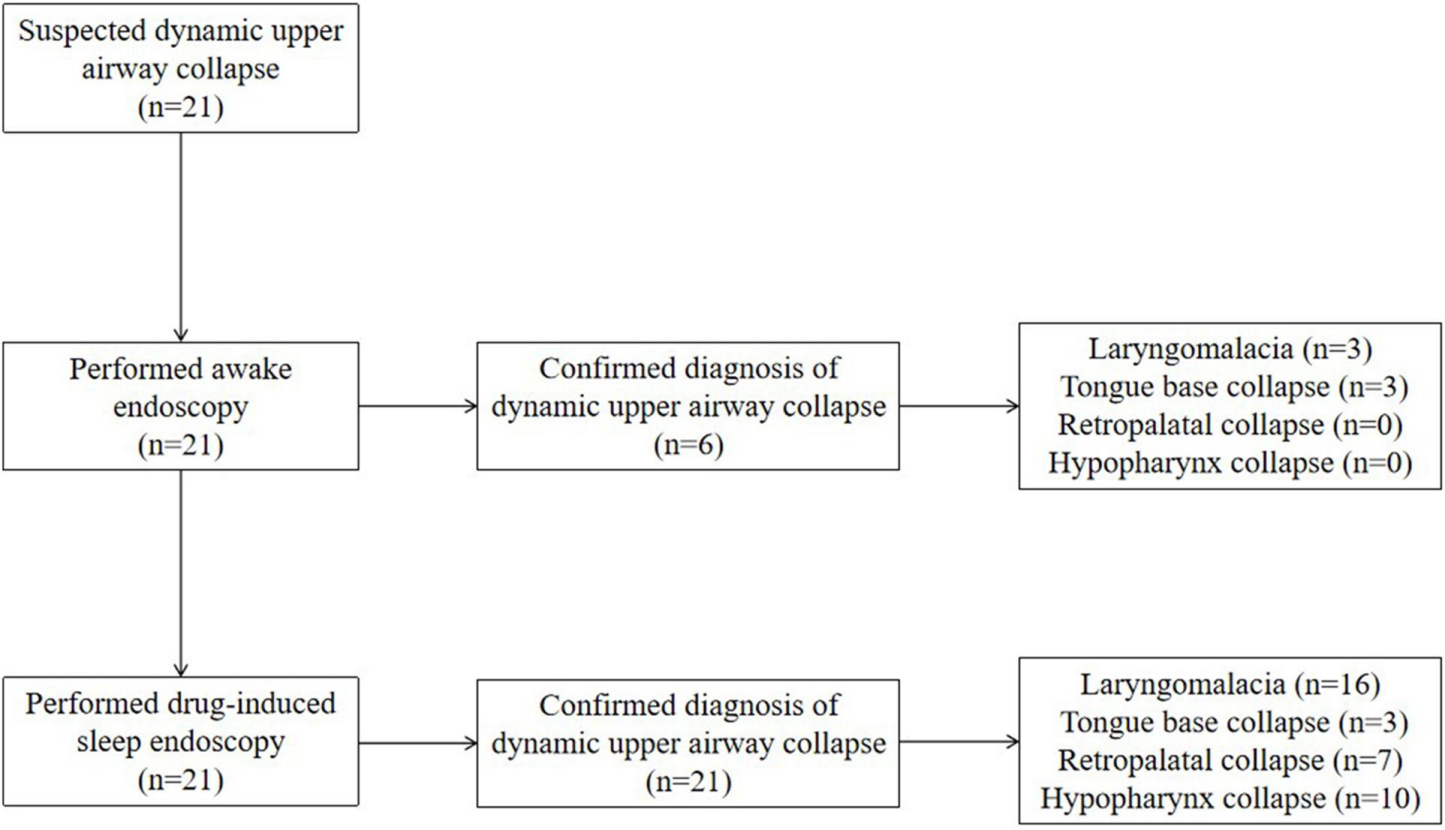
The study flow diagram and endoscopic findings.

A total of 21 cases (male *n* = 17, female *n* = 4) were included. All 21 cases were initially suspected of having dynamic upper airway collapse based on clinical manifestations and were definitively diagnosed by endoscopy. The study flow diagram is shown in Figure 1. The cohort included 3 neonates and 18 cases beyond neonatal age. Six cases were born preterm, and 15 cases were born full term. At the time of bronchoscopy, the median age was 4.0 (2.0–9.0) months, and the median weight was 5.5 (4.2–7.4) kg. In terms of clinical manifestations, noisy breathing was observed in 12 cases, suprasternal retraction in 8 cases, and snoring in 7 cases (Table 1).

**Table 1:** Clinical data and endoscopy of the cases with dynamic upper airway collapse.

No.	Gender	Sedation strategy	Age at the time of endoscopy	Weight at the time of endoscopy	Clinical manifestations	Endoscopy during AE	Clinical manifestations during DISE	Endoscopy during DISE
1	M	Dexmedetomidine 4 µg/kg, midazolam 0.2 mg/kg	10 months	5.4 kg	Suprasternal retraction	Normal	Stridor, exacerbation of suprasternal retraction	PAC (lateral hypopharynx collapse), laryngomalacia (type Ⅰ + Ⅱ + III)
2	F	Midazolam 0.2 mg/kg	13 months^a^	8.4 kg	Noisy breathing during activity and sleep	Normal	Noisy breathing, suprasternal retraction	PAC (lateral retropalatal collapse + lateral hypopharynx collapse)
3	M	Midazolam 0.2 mg/kg	8 months	6.8 kg	Noisy breathing during activity	Normal	Suprasternal retraction	Laryngomalacia (type Ⅱ + III)
4^d^	M	10% chloral hydrate 0.5 mL/kg	Neonate	3.4 kg	Suprasternal retraction	Normal	Exacerbation of suprasternal retraction	PAC (lateral hypopharynx collapse), laryngomalacia (type III)
5^d^	M	Midazolam 0.3 mg/kg	13 months^a^	9.2 kg	Noisy breathing during activity	Normal	Noisy breathing, suprasternal retraction	PAC (lateral hypopharynx collapse), laryngomalacia (type Ⅱ + III)
6	M	Midazolam 0.1 mg/kg	8 months	5.7 kg	Noisy breathing during activity and sleep	Normal	Noisy breathing, suprasternal retraction	Laryngomalacia (type Ⅰ + Ⅱ)
7	M	Midazolam 0.2 mg/kg	2 months	5.5 kg	Snoring	Normal	Snoring, suprasternal retraction	PAC (lateral retropalatal collapse)
8^d^	M	Midazolam 0.1 mg/kg	4 months	6.0 kg	Noisy breathing	Laryngomalacia (type Ⅱ + III)	Noisy breathing, suprasternal retraction	Laryngomalacia (type Ⅱ + III)
9^d^	M	Midazolam 0.1 mg/kg	5 months	5.4 kg	Stridor, suprasternal retraction	Laryngomalacia (type Ⅰ + Ⅱ + III)	Exacerbation of stridor and suprasternal retraction	Laryngomalacia (type Ⅰ + Ⅱ + III)
10	M	Midazolam 0.2 mg/kg	5 months	7.4 kg	Snoring	Normal	Snoring, suprasternal retraction	PAC (lateral retropalatal collapse)
11	M	10% chloral hydrate 0.5 mL/kg, phenobarbital 5 mg/kg	Neonate	3.5 kg	Snoring, suprasternal retraction	Normal	Snoring, exacerbation of suprasternal retraction	PAC (lateral hypopharynx collapse)
12^d^	M	Midazolam 0.2 mg/kg	2 months	4.0 kg	Noisy breathing, snoring	PAC (tongue base collapse)	Snoring, suprasternal retraction	PAC (lateral hypopharynx collapse + tongue base collapse)^c^, laryngomalacia (type Ⅰ)
13	M	Midazolam 0.2 mg/kg	4 months	7.4 kg	Noisy breathing during activity, snoring	Normal	Stridor, suprasternal retraction	PAC (lateral hypopharynx collapse), laryngomalacia (type Ⅰ + Ⅱ)
14	M	Dexmedetomidine 4 µg/kg, midazolam 0.3 mg/kg	11 months	8.2 kg	Noisy breathing	Normal	Noisy breathing, suprasternal retraction	PAC (anteroposterior retropalatal collapse + lateral hypopharynx collapse)
15	F	Midazolam 0.2 mg/kg	4 months	4.2 kg	Stridor, suprasternal retraction	Laryngomalacia (type Ⅰ + Ⅱ + III)	Stridor, suprasternal retraction	Laryngomalacia (type Ⅰ + Ⅱ + III)
16	M	10% chloral hydrate 0.5 mL /kg	Neonate	4.1 kg	Noisy breathing, suprasternal retraction	PAC (tongue base collapse)	Exacerbation of suprasternal retraction	PAC (tongue base collapse), laryngomalacia (type III)
17^d^	M	Midazolam 0.2 mg/kg	10 months	5.0 kg	Suprasternal retraction	Normal	Exacerbation of suprasternal retraction	Laryngomalacia (type Ⅰ)
18^b^	F	Midazolam 0.2 mg/kg	1 months	4.0 kg	Noisy breathing, snoring, suprasternal retraction	PAC (tongue base collapse)	Stridor, exacerbation of suprasternal retraction	PAC (lateral retropalatal collapse + lateral hypopharynx collapse + tongue base collapse)^c^, laryngomalacia (type Ⅰ + Ⅱ)
19	M	Midazolam 0.1 mg/kg	3 months	5.0 kg	Noisy breathing during activity	Normal	Stridor, suprasternal retraction	PAC (lateral retropalatal collapse), laryngomalacia (type Ⅰ + Ⅱ + III)
20	M	Midazolam 0.2 mg/kg	4 months	7.3 kg	Snoring	Normal	Stridor, suprasternal retraction	PAC (lateral hypopharynx collapse), laryngomalacia (type Ⅰ + Ⅱ)
21	F	Midazolam 0.3 mg/kg	5 months	6.4 kg	Noisy breathing during activity	Normal	Suprasternal retraction	PAC (lateral retropalatal collapse), laryngomalacia (type III)

M, male; F, female; PAC, pharyngeal airway collapse.

^a^ Cases with clinical manifestations of dynamic upper airway collapse at 1-year-old.

^b^ Cases that developed a hypoxic episode during DISE, which was resolved by pressurized facial mask-assisted ventilation.

^c^ Exacerbation of tongue base collapse was found in the cases during the inspiratory period under DISE compared with AE.

^d^ Cases born preterm, and the rest born full term.

### Sedation strategies during DISE

For the cases beyond neonatal age (*n* = 18), 16 (88.9%) received midazolam only, and 2 (11.1%) received midazolam combined with dexmedetomidine. For the neonates (*n* = 3), two (66.7%) received 10% chloral hydrate only, and one (33.3%) received 10% chloral hydrate combined with phenobarbital. No cases received propofol. All cases successfully completed DISE (Table 1).

### Endoscopic findings

Laryngomalacia was diagnosed in 16 cases (76.2%), and PAC was diagnosed in 15 cases (71.4%). Ten cases (47.6%) concurrently had laryngomalacia and PAC. Of the cases with laryngomalacia, 5 (31.3%) had a single type of laryngomalacia, and 11 (68.7%) had a mixed type. Of the cases with PAC, 3 (20.0%) had tongue base collapse, 7 (46.7%) had retropalatal collapse, and 10 (66.7%) had hypopharynx collapse, whereas 4 (26.7%) had multilevel collapse (Figure 1 and Table 1).

### Comparisons between AE and DISE

**Figure 2:**
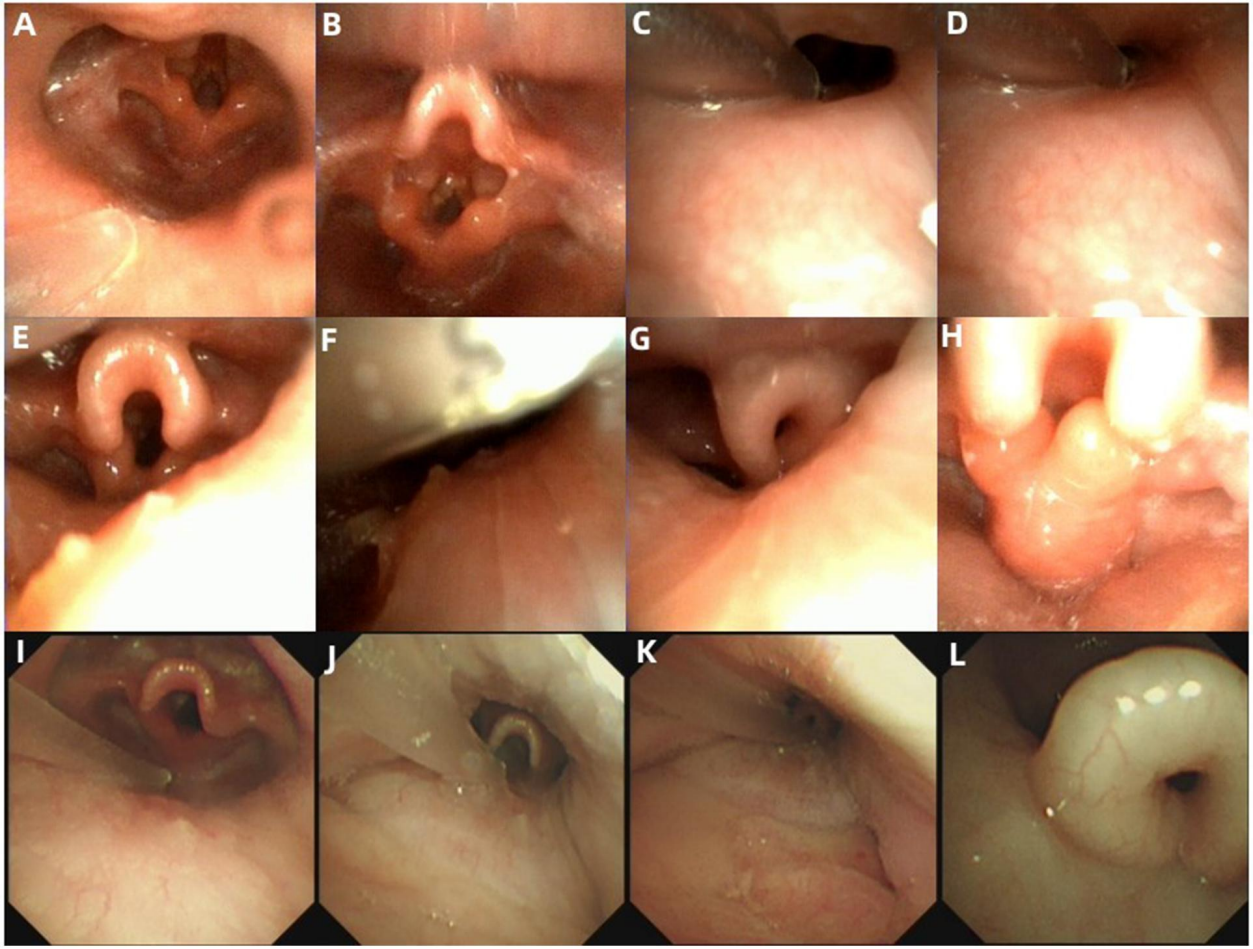
Endoscopic presentations in the cases with dynamic upper airway collapse. **(A–H)** Endoscopic findings in Case 18. **(A)** Normal appearance of the retropalatal cavity during the inspiratory period under AE. **(B)** Tongue base collapse during the inspiratory period under AE. **(C)** Retropalatal cavity during the expiratory period under DISE. **(D)** A significant reduction of retropalatal internal diameter during the inspiratory period under DISE. **(E)** Hypopharyngeal cavity during the expiratory period under DISE. **(F)** A significant reduction of the hypopharyngeal internal diameter during the inspiratory period under DISE. **(G)** Epiglottis curling and exacerbation of tongue base collapse during the inspiratory period under DISE. **(H)** Inward collapse of the redundant arytenoid mucosa and cartilages during the inspiratory period under DISE. **(I–L)** Endoscopic findings in Case 5. **(I)** Normal appearance of the hypopharyngeal cavity during the inspiratory period under AE. **(J)** Hypopharyngeal cavity during the expiratory period under DISE. **(K)** A significant reduction of the hypopharyngeal internal diameter during the inspiratory period under DISE. **(L)** Epiglottis curling during the inspiratory period under DISE.

Of the cases with dynamic upper airway collapse, 6 (28.6%) were diagnosed under both awake endoscopy (AE) and DISE, whereas 15 (71.4%) were diagnosed under DISE only. The diagnostic rate was significantly higher under DISE than that under AE (100.0% vs. 28.6%, *P* < 0.01). Of the cases with laryngomalacia, 3 (18.7%) were diagnosed under both AE and DISE, whereas 13 cases (81.3%) were diagnosed under DISE only. The diagnostic rate was significantly higher under DISE than that under AE (100.0% vs. 18.7%, *P* < 0.01). Of the cases with tongue base collapse, all (100.0%) were diagnosed under both AE and DISE, whereas two (66.7%) presented exacerbation of the collapse under DISE compared with AE. Of the cases with retropalatal collapse and hypopharynx collapse, all (100.0%) were diagnosed under DISE only (Figure 2 and Table 1).

### Adverse events during DISE

One case (4.8%) developed a hypoxic episode during DISE, which was resolved by pressurized facial mask-assisted ventilation (Table 1).

## Discussion

Dynamic upper airway collapse is a group of diseases characterized by narrowing of the upper airway lumen and airflow limitation during inspiration. In adults, it typically presents as snoring and OSA (11). Since abnormal performance mainly occurs during sleep, DISE is widely used in adults to evaluate the dynamic upper airway collapse, which can help make the treatment decision and predict surgical outcomes. However, the presentation differs in children especially in infants. In addition to snoring and OSA during sleep, infants with dynamic upper airway collapse usually present with stridor or noisy breathing during wakefulness (4). This suggests that AE, rather than DISE, might also display the abnormal performance and help diagnose dynamic upper airway collapse in infants. Moreover, sedative or anesthetic drugs used during DISE might worsen airway obstruction in infants with dynamic upper airway collapse (12). Therefore, the role of DISE in the diagnosis of dynamic upper airway collapse in infants remains controversial. In view of this, we conducted this study to evaluate the utility and safety of DISE in infants with suspected dynamic upper airway collapse.

In this study, dynamic upper airway collapse was defined as the presence of PAC and/or laryngomalacia. In infants, PAC is often missed during endoscopic evaluation (13). This is partly due to a lack of awareness of the condition (4). Another reason may be poor compliance in infants. Thus far, few studies have reported an endoscopic diagnostic method for PAC in infants. During AE, infants usually fuss and cry, and when they do this, the pharyngeal isthmus closes due to soft palate elevation, making it difficult to assess retropalatal collapse (3, 14). DISE can eliminate the fussing and crying during endoscopy, allowing better diagnosis of retropalatal collapse. In this study, all cases with retropalatal collapse were diagnosed under DISE only. It was noteworthy that all cases with hypopharynx collapse were also diagnosed under DISE only, demonstrating that the hypopharyngeal cavity may maintain patency during wakefulness. Therefore, identification of hypopharynx collapse may also rely on DISE. Moreover, laryngomalacia in infants may be sleep-dependent (15), requiring DISE to make a diagnosis. In this study, 14 cases with laryngomalacia were confirmed, 11 of which were sleep-dependent. In view of the above reasons, DISE appeared to improve the diagnostic rate for dynamic upper airway collapse in infants compared with AE. The results of this study support this viewpoint. In this study, the majority of the cases (68.4%) with dynamic upper airway collapse revealed abnormal under DISE only but revealed normal under AE. Furthermore, the diagnostic rate was significantly higher under DISE than that under AE in cases with dynamic upper airway collapse.

The choice of sedative or anesthetic drugs for pediatric DISE is still controversial. The ideal sedative or anesthetic drugs for pediatric DISE are those that can mimic natural sleep with a wide safety margin (2). In this study, we chose dexmedetomidine and/or midazolam for DISE in cases beyond neonatal age, and all cases had completed DISE successfully. At present, midazolam and dexmedetomidine, either alone or in combination, are considered optimal sedation or anesthetic agents for pediatric DISE (2). In fact, propofol is also considered suitable for DISE (2). However, the major concerns included its potential for dose-dependent airway collapse and the need for target-controlled infusion, as rapid infusion can cause central sleep apnea (16). In view of this, propofol was not used in this study. In addition, ketamine is also avoided in pediatric DISE as it can increase muscle tone, which can stiffen the airway and mask a true obstructive breathing pattern (2). Inhalational agents are also not suitable for pediatric DISE, as they can cause upper airway obstruction in a dose-dependent manner (2). In this study, neither ketamine nor an inhalational agent was used for DISE. It is worth noting that there have been no reports of DISE performed in neonates until now. Therefore, the choice of sedative or anesthetic drugs for neonatal DISE is typically inexperienced. Due to safety concerns, we used 10% chloral hydrate—combined with phenobarbital or not—for neonates, all of whom also completed DISE successfully.

The safety of DISE in infants with dynamic upper airway collapse warrants focused attention. Some infants with dynamic upper airway collapse may develop hypoxic episodes due to upper airway obstruction (4). It is well known that sedative or anesthetic drugs can induce sleep and reduce the pharyngeal muscle tension, which can aggravate upper airway obstruction (12). In this study, one case (4.8%) developed a hypoxic episode during DISE, which was resolved by pressurized facial mask-assisted ventilation. Therefore, application of DISE in infants with dynamic upper airway collapse is safe and feasible. However, the emergency planning for hypoxic episodes during DISE should be well prepared.

This study has some limitations. First, it was a single-center study with a small sample size. Second, this study had a certain bias in case selection. All cases in this study were examined using a thin bronchoscope with an outer diameter of 2.8 mm. However, in very small infants, particularly preterm neonates, this size may still be relatively large for the airway. Moreover, the cases that were unable to tolerate the sedative or anesthetic drugs were excluded. These factors may influence both the safety and diagnostic accuracy of dynamic airway collapse assessment. Third, this was a self-control study without a control group of infants without dynamic upper airway collapse, limiting the ability to further identify potential misdiagnosis by DISE. Multicenter studies with larger sample sizes should be performed in the future.

## Conclusions

DISE was found to be feasible and safe in infants with suspected dynamic upper airway collapse. Compared with AE, DISE improved the diagnostic rate of laryngomalacia and appeared to be a more reliable method for diagnosing PAC, especially retropalatal and hypopharynx collapse.

## Data Availability

The original contributions presented in the study are included in the article/Supplementary Material, further inquiries can be directed to the corresponding authors.
